# Histone deacetylases inhibition by SAHA/Vorinostat normalizes the glioma microenvironment via xCT equilibration

**DOI:** 10.1038/srep06226

**Published:** 2014-09-17

**Authors:** Ines M. L. Wolf, Zheng Fan, Manfred Rauh, Sebastian Seufert, Nirjhar Hore, Michael Buchfelder, Nic E. Savaskan, Ilker Y. Eyüpoglu

**Affiliations:** 1 Department of Neurosurgery, Universitätsklinikum Erlangen, Friedrich Alexander Universität Erlangen-Nürnberg (FAU); 2 Department of Pediatrics and Adolescent Medicine, University of Erlangen-Nuremberg; 3 Institute of Human Genetics, University of Cologne; 4 These authors contributed equally to this work.; 5 These authors jointly supervised this work.

## Abstract

Malignant gliomas are characterized by neurodegenerative actions leading to the destruction of surrounding brain parenchyma. The disturbance in glutamate homeostasis caused by increased expression of the glutamate transporter xCT plays a key role in glioma progression. We demonstrate that the HDAC-inhibitor SAHA specifically inhibits the xCT-transporter expression. Thereby, tumor cell stress is engendered, marked by increase in ROS. Moreover, SAHA dependent xCT-reduction correlates with the inhibition of ATF4-expression, a factor known to foster xCT expression. Since xCT/system Xc- is pivotal for the brain tumor microenvironment, normalization of this system is a key in the management of malignant gliomas. To date, the problem lay in the inability to specifically target xCT due to the ubiquitous expression of the xCT-transporter—i.e. in non-cancerously transformed cells too—as well as its essential role in physiological CNS processes. Here, we show xCT-transporter equilibration through SAHA is specific for malignant brain tumors whereas SAHA does not affect the physiological xCT levels in healthy brain parenchyma. Our data indicate that SAHA operates on gliomas specifically via normalizing xCT expression which in consequence leads to reduced extracellular glutamate levels. This in turn causes a marked reduction in neuronal cell death and normalized tumor microenvironment.

The acetylation and deacetylation of histones represent important epigenetic regulatory mechanisms of gene expression^1^,^2^,^3^. Whereas acetylation of histones leads to an unfolding of chromatin structures and promotes increased gene transcription, deacetylation is associated with chromatin condensation and mediates suppression of gene activity^4^,^5^,^6^. These processes are accomplished by the balanced activities of histone acetyltransferases (HAT) and histone deacetylases (HDAC). A normal, physiological cell differentiation as well as an appropriate cellular metabolic activity mandates a fine tuned regulation of the respective HATs with regard to the HDACs. Correspondingly, a misbalance between HAT and HDAC is subsequently associated with oncogenic transformation processes^7^,^8^. In this context, the option of blocking the activity of HDACs through specific small molecule inhibitors opens up new avenues of pharmacological intervention in gene transcription through epigenetic regulation. Furthermore, HDAC-inhibitors (HDACi) have the particular property of specific effects on tumor cells^9^. As opposed to conventional chemotherapeutic agents, they do not act globally on the entire parenchyma. Instead, HDAC-inhibitors operate selectively on transformed cells^10^,^11^. A fundamental mechanism behind this selectiveness is considered in the balance between thioredoxin and reactive oxygen species (ROS)^9^. This will permit counter-effecting the described oncological dysbalance and gain significance as a new treatment concept for patients suffering from glioblastoma (WHO °IV) where treatment options are currently sparse. Glioblastomas, with a median survival time of just 14 months belong to one of the most malignant tumor entities altogether^12^. They belong to the group of malignant gliomas, which at 70% represent by far the most frequently occurring primary brain tumors. The poor prognosis is primarily attributed to heightened proliferation, diffuse invasion^13^, enhanced angiogenesis^14^, suppression of immune competent cells^15^,^16^ and the ability to effect need-oriented transformation of the tumor microenvironment^17^. These properties are arrived at primarily through the production and secretion of factors which manipulate the microenvironment to the advantage of the tumor^18^. One of these factors is the amino-acid glutamate. It is expelled out of tumor cells in exchange for cysteine through the antiporter xCT (also known as system Xc-, encoded by SLC7a11 in humans). Importantly, xCT expression leads to an increase in chemo-resistance^19^. Furthermore, gliomas-derived extracellular glutamate levels are significantly involved in the induction of perifocal edema^17^. Additionally, glutamate stimulates microglial activity and leads to their accumulation^20^,^21^, which can additively trigger neuronal cell death through dendritic retraction^22^. The phagocytic microglial activity is subsequently paralyzed by tumor cells and an immune response to the neoplastic process is absent^15^. Furthermore, levels of extracellular secreted glutamate is toxic and causes neuronal cell death, through which room for unrestricted tumor growth is created^23^. The regulation of the extracellular glutamate content therefore occupies a key position in the progression of malignant gliomas. For clinical neuro-oncology, these glioma features mean that in the face of lack of a cure, an imminent tumor recurrence must be reckoned with in almost certainty. On the other hand, such characteristics represent an ideal fulcrum for treatment with HDAC-inhibitors, as it is to be assumed that HDAC-inhibitors can decisively influence precisely these factors^24^,^25^. A possible growth-inhibiting mode of action in glioblastomas is the specific up-regulation of the cell cycle control protein p21/WAF^23^. In this manner, tumor cells are lead to a G0-G1 cell cycle arrest and consecutively to apoptotic cell death^26^. Not only do HDAC-inhibitors directly influence the tumor, they also lead to a readjustment of the tumor microenvironment^27^,^28^ accompanied by a reduction in glioma-induced neuronal cell death^23^. We therefore assume that HDAC-inhibitors can act in a regulatory manner on the decompensated glutamate metabolism in malignant gliomas, which we demonstrate in this study for the first time.

## Results

### SAHA specifically suppresses the expression of the glutamate transporter xCT

**Figure 1:**
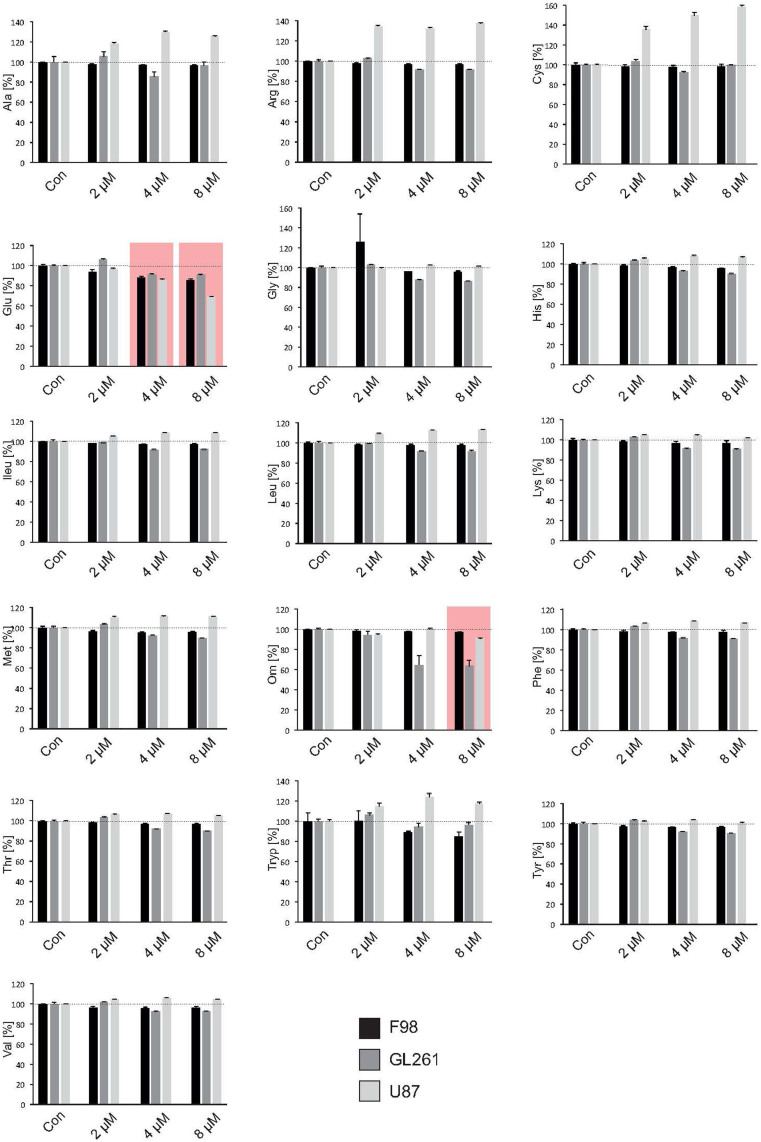
SAHA reduces glutamate secretion. The amino acid secretome in gliomas cells was assessed by fully automatized ion-exchange chromatography analysis. Three well established glioma cell lines (human U87, rat F98, mouse GL261) were facilitated for this assay. The amino-acid serine was used as a reference and the glutamate/serine quotient calculated. SAHA silenced xCT-expression with an attendant, significant decrease in extracellular glutamate levels in all 3 cell lines already at 4 μM. Accordingly, there was no change in the extracellular concentration of the amino-acid cysteine. Statistical significance was calculated with the Student's t-test (mean ± sem, *P < 0.05, n = 3 per group).

**Figure 2:**
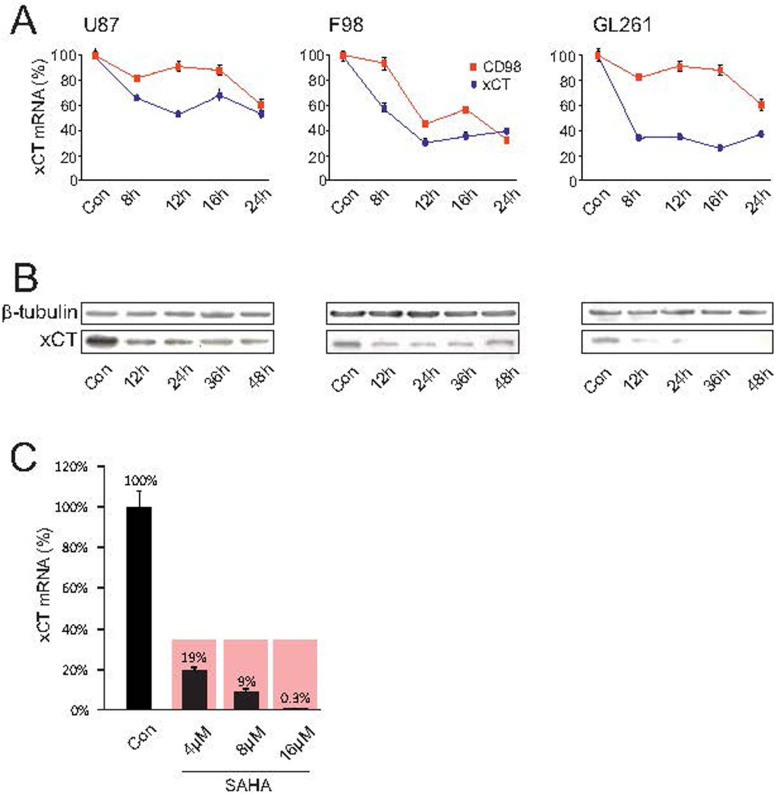
SAHA silences xCT expression and function. (A), The human U87MG, rat F98 and mouse GL261 glioma cell lines were treated with 8 μM SAHA and xCT as well as CD98 expression were subsequently analyzed by real-time RT-PCR. A significant suppression of xCT was observed in all three established glioma cell lines. (B), xCT expression analysis by immunoblotting. Down-regulation of xCT protein was observed after treatment with 8 μM SAHA already 12 hours following treatment. Membranes were reprobed for β-tubulin expression to show that similar amounts of protein were loaded in each lane. The cropped blots are shown in the figure and the full-length blots are presented in supplementary figure 1. (C), SAHA inhibits xCT expression in human malignant glioma tissue. Glioblastoma tissue obtained from a GBM patient was cultivated and treated with 4 μM SAHA, leading to a significant reduction of xCT-expression. Statistical significance was calculated with the Student's t-test (mean ± sem, *P < 0.05, n = 3 per group).

The inhibitor SAHA modulates gene-expression by blocking specifically the HDAC classes I, IIa, IIb and IV. We investigated the extent to which this pan HDAC inhibitor also affects the expression of various target genes playing a decisive role in shaping the tumor microenvironment. To this end, we analyzed the metabolic profile for 3 different glioma cell lines (U87 human, F98 rat, GL261 mouse) under SAHA treatment and quantified 16 secreted amino-acids (alanin, arginine, cysteine, glutamate, glycine, histidine, isoleucine, leucine, lysine, methionine, ornithine, phenylalanine, threonine, tryptophan, tyrosine, valine) by means of liquid chromatography-mass spectrometry (LC-MS) (Fig. 1). The amino-acid serine was used as a reference to calculate the glutamate/serine quotient. Only the secretion of the two amino-acids glutamate and ornithine were uniformly reduced in all 3 cell lines, with significant reduction in glutamate levels already seen at the low concentration of 4 μM SAHA (Fig. 1). We could observe that the expression of the glutamate transporter xCT/system Xc- (comprising of xCT and CD98) is significantly suppressed already at low concentrations. At 8 μM SAHA, the xCT- or CD98 expressions was altered in three tested glioma cell lines human U87, rat F98 and mouse GL261 following 8 hours treatment, which could be demonstrated by real-time RT-PCR (Fig. 2A). We verified these findings assessing the protein levels. Here, too, 8 μM SAHA was sufficient to suppress xCT-expression, which was confirmed by Western blot for xCT following 12 hours treatment (Fig. 2B).

**Figure 3:**
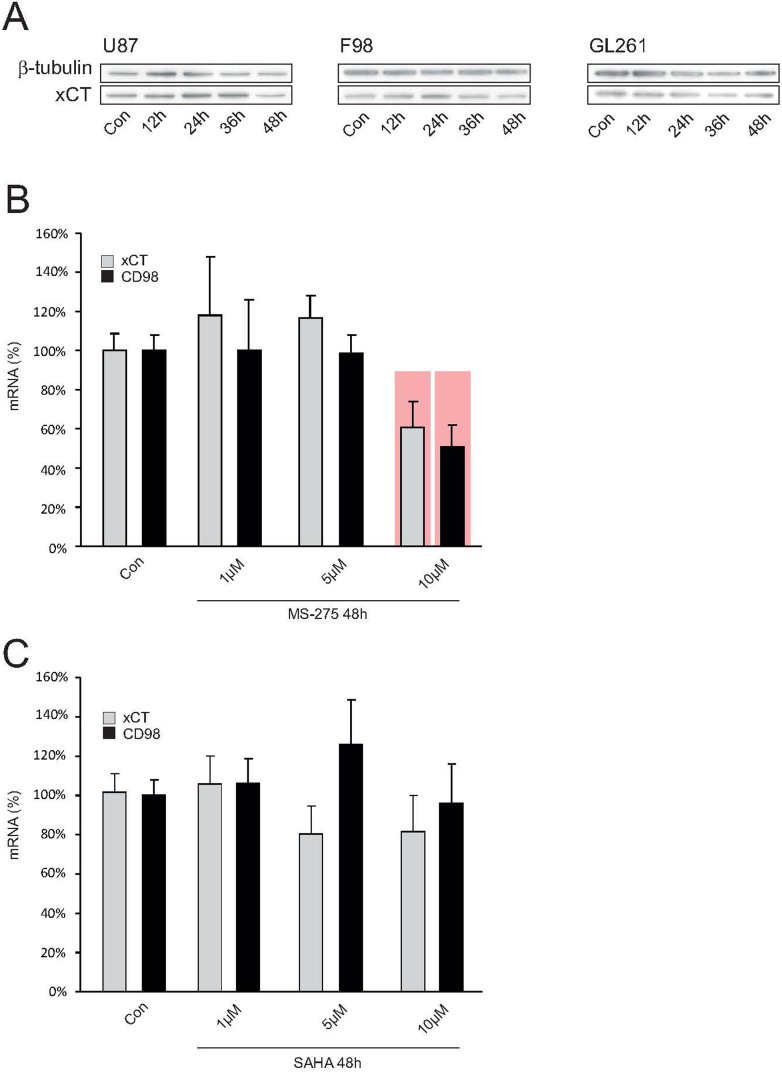
HDAC inhibitor SAHA acts specifically on xCT expression. (A), The HDAC-inhibitor MS-275 does not affect xCT expression. MS-275 is a specific inhibitor of HDACs class I and was further evaluated for its ability to silence xCT-expression by Western blot. No regulation of xCT-expression was observed after treatment with MS-275 in the cell lines human U87MG, rat F98 and mouse GL261. Membranes were reprobed for β-tubulin expression to show that similar amounts of protein were loaded in each lane. The cropped blots are shown in the figure and the full-length blots are presented in supplementary figure 2. (B), MS-275 affects xCT in healthy brain tissue. The effect of MS-275 was also analyzed in healthy brain slice-cultures from rat with real-time RT-PCR. Here, only at 10 μM MS-275 a significant suppression of xCT and CD98 could be detected. (C), SAHA does not challenge xCT expression in healthy brain tissue. To further investigate the specificity with which the HDAC-inhibitor SAHA selectively modulates tumor cells and whether it incurs any adverse effects in healthy brain tissue, slice-cultures from healthy rat brains were treated with SAHA and the xCT- and CD98-expressions were analyzed again with real-time RT-PCR. No significant changes in xCT-expression were detectable. Thus, SAHA selectively changes xCT-expression in malignant gliomas, while healthy brain parenchyma remains unaffected by this HDAC-inhibitor. Statistical significance is calculated with the Student's t-test (mean ± s.d., *P < 0.05, n ≥ 8 per group).

Although we could establish that SAHA modulates xCT-expression through SAHA in the human glioma cell line U87, we further tested how far these results could be extrapolated to human biology through an independent approach. For this we cultured human glioblastoma specimens taken from one GBM patient and treated the human tissue with SAHA. We found that the suppressive effects here were even more pronounced than in the experiments with the cell lines. Already 4 μM SAHA reduced xCT-expression to 19% in comparison to the control group (Fig. 2C). The effect of SAHA on xCT was even more potent: 8 μM suppressed xCT-expression to 9% and following 16 μM the xCT-expression was almost beyond the detection level at 0.3 μM (Fig. 2C). In order to test the extent to which this HDAC-inhibitor is specific in regulating the glutamate transporter system Xc-, we tested another SAHA–independent HDAC inhibitor named MS-275. To this end, xCT-expression following treatment with 8 μM MS-275 in the cell lines human U87, rat F98 and mouse GL261 was analyzed with Western blot. Here, we did not find any alterations and xCT-expression remained unchanged (Fig. 3A). MS-275 caused no change in xCT expression in malignant gliomas. In contrast, MS-275 altered xCT levels in healthy brain parenchyma, indicating that MS-275 exhibits general toxic effects (Fig. 3B). Further, only limited modulation of the xCT-transporter could be induced in healthy brain tissue. Organotypic slice-cultures from rat brains were then treated with 8 μM MS-275 for 48 hours and xCT- and CD98-expressions subsequently analyzed with real-time RT-PCR. Only at 10 μM MS-275 a significant suppression of the xCT-transporter could be detected, indicating a global effect on gene expression (Fig. 3B). To further investigate the specificity with which the HDAC-inhibitor SAHA selectively modulates tumor cells and whether it incurs any adverse effects in healthy brain tissue, organotypic slice-cultures from healthy rat brains were treated with SAHA and the xCT- and CD98-expressions were analyzed again with real-time RT-PCR. Here, too, no significant changes in xCT-expression were noted (Fig. 3C). Thus, SAHA selectively changes xCT-expression in malignant gliomas, while healthy brain parenchyma remains unaffected by this particular HDAC-inhibitor.

### SAHA reduces xCT-expression through inhibition of ATF4

**Figure 4:**
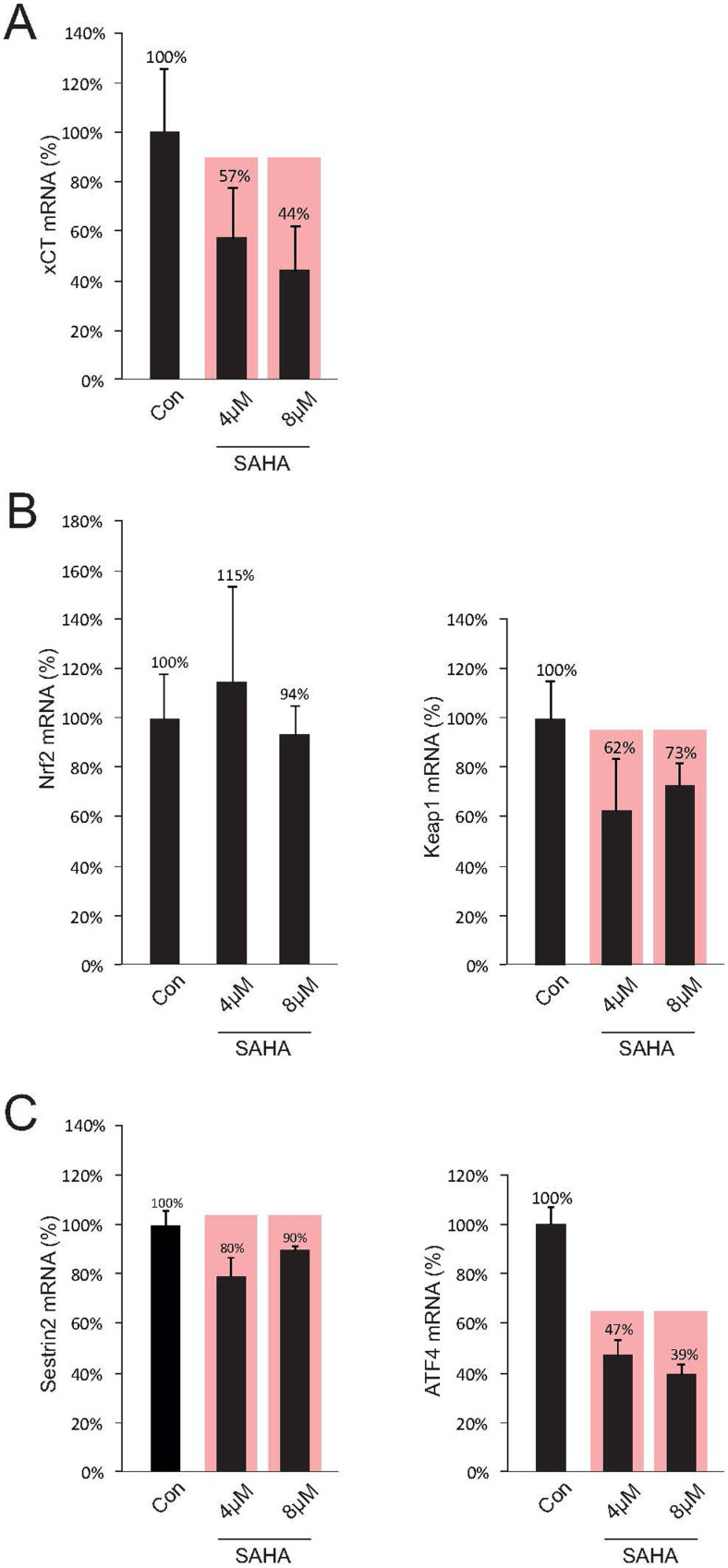
SAHA affects ATF4. (A), Treatment with SAHA results in a significant suppression of xCT-expression, which was detected by real-time RT-PCR. (B), The same probes were then utilized to analyze the factors Nrf2 and Keap1 through real-time RT-PCR. SAHA reduces significantly the expression of Keap1, while Nrf2 is unaffected. (C), The expression of sestrin2 is marginally but significantly reduced after SAHA treatment, while the xCT regulating transcription factor ATF4 is reduced in its expression following SAHA treatment. Since changes in sestrin2 expression were both weak and dosage-independent, it can be assumed that SAHA does not regulate xCT expression mainly through the Sestrin2/Keap1/Nrf2 axis. Statistical significance was calculated with the Student's t-test (mean ± s.d., *P < 0.05, n ≥ 8 per group).

The expression of the xCT-transporter is primarily controlled through the transcription factors Keap1 and Nrf2, where Keap1 binds Nrf2 and only free Nrf2 induces xCT-protein transcription^29^,^30^,^31^. A superordinate system here is represented by the sestrines, where sestrin2 significantly controls the Nrf2/Keap1 system by setting Nrf2 free to activate the xCT-promoter through degrading Keap1^32^. In a further step we analyzed the expression levels of these factors under SAHA treatment and investigated the impact by quantitative RT-PCR. Initially, we have demonstrated that the xCT-expression could be reduced significantly to 57% below control levels under 4 μM SAHA and at 8 μM SAHA down to 44% (Fig. 4A). The same samples were then utilized for analyzing the transcription factors Nrf2 and Keap1. We could clearly show that SAHA treatment does not directly affect Nrf2 since reduction of Nrf2-expression under SAHA could not be detected (Fig. 4B, left row). However, a significant reduction in Keap1 to 62% following treatment with 4 μM SAHA and to 73% following 8 μM SAHA was achieved under these conditions (Fig. 4B, right row). The expression of sestrin2 could be reduced to 80% after 4 μM SAHA and to 90% following 8 μM (Fig. 4C, left row). Independent of this signal pathway, xCT-expression is additionally regulated by the transcription factor ATF4^33^. The activating ATF4-mutation here leads to an increase in xCT-expression in tumor cells and reduction in ATF4 levels is conversely associated with a diminished xCT-expression. These findings could be confirmed by our own investigations. 4 μM SAHA led to a significant reduction in ATF4-expression to 47% of control levels, whereas 8 μM suppressed the expression even up to 39%. (Fig. 4C, right row). These results allow the assumption that massive xCT suppression under SAHA treatment is mainly dependent on the expression levels of ATF4 rather than the sestrin2 signal pathway.

### SAHA reduces devastating effects of gliomas in the tumor microenvironment

**Figure 5:**
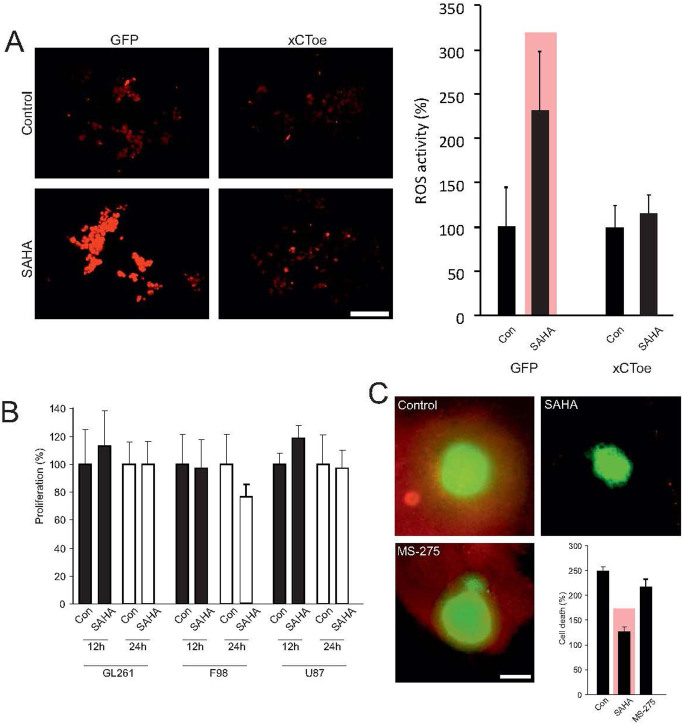
SAHA alleviates xCT-dependent cell death. (A), Treatment of F98 glioma cells with SAHA is associated with tumor cell stress, as measured by ROS-accumulation. Quantification of ROS levels shows a significant increase in ROS after SAHA treatment. Stable over-expression of the xCT transporter leads to silencing of this effect and ROS activation through SAHA was no longer in evidence. These data show that SAHA induced suppression of the xCT transporter specifically lead to ROS activation, thereby resulting in cell stress. (B), However, the described cell stress was not accompanied by growth inhibition, which is analyzed by MTT assay. (C), F98 glioma cells were implanted on living organotypic brain slices and tumor-induced cell death analyzed through live imaging. Malignant gliomas induced neuronal cell death in surrounding healthy brain parenchyma, while treatment with the HDAC-inhibitor SAHA blocked neurodegeneration completely. Corresponding treatment with the HDAC inhibitor MS-275 does not lead to neuroprotection. Statistical analysis of neuronal cell death shows a significant reduction in PI positive cells after treatment with SAHA, whereas treatment with MS-275 did not achieve this effect. Statistical significance is calculated with the Student's t-test (mean ± s.d., *P < 0.05, n ≥ 6 per group).

Malignant gliomas secrete glutamate into the extracellular space via the xCT-transporter. In exchange, cysteine is taken up into tumor cells via the same transporter as antiport^34^. Further, suppressed transporter expression leads to reduced glutamate export and levels in the extracellular space, and glutamate accumulates intracellular in tumor cells. Moreover, cysteine necessary for intracellular detoxification processes is lacking^35^. Correspondingly, the extracellular concentration of cysteine remained not altered or even increased in comparison to controls (Fig. 1). This implies that reduced xCT-expression should be accompanied by tumor cell stress and thereby with an accumulation of reactive oxygen species (ROS). Therefore, to test this we treated glioma cells with 8 μM SAHA and subsequently analyzed ROS levels as an assay to monitor cell stress (Fig. 5A, left row). Here, we observed a significant elevation of ROS levels from 100% to 232% (Fig. 5A, right row). In order to examine the extent to which the rise in ROS was directly caused by the inhibition in xCT expression, we generated a F98 glioma cell line with increased xCT expression. Analysis of the ROS activity following treatment of these cells with 8 μM SAHA showed no significant ROS increase in comparison to controls (Fig. 5A). These data indicate that SAHA suppresses xCT expression leading to a specific increase in ROS activity. Since SAHA can also suppress the proliferation of tumor cells, we investigated the extent to which this applied to analyses in a further step. Our observations were carried out within a time-frame of 24 hours following SAHA-treatment, and hence analyzed tumor growth through MTT assay in three different glioma cell lines within the same time-frame. However, no change in tumor growth was observed during 12 and 24 hours incubation with 8 μM SAHA in comparison to controls (Fig. 5B).

A further consequence of blocked glutamate secretion lies in the absence of glutamate-associated changes in the tumor microenvironment, where primarily the aquaporin mediated astrocyte swelling as well as the peritumoral edema are suppressed^17^. Additionally, glutamate can induce excitatory neuronal cell death through NMDA receptor activation^20^. The extent to which this applies to glioma-associated glutamate secretion in this context is hitherto unclear. We have therefore implanted glioma cells on living organotypic brain slices and analyzed tumor-induced cell death by means of live cell imaging^23^. Malignant gliomas have the ability to induce neuronal cell death in surrounding healthy brain parenchyma. Treatment with the HDAC-inhibitor SAHA protected the surrounding, vital brain parenchyma from cell death (Fig. 5C). Tumor implantation affected a raise in cell deaths from 100% to 248% in comparison to glioma free brain slices. When we applied SAHA to brain tissue cell death was significantly reduced to 126% by SAHA (Fig. 5C). The extent to which the observed neuroprotective effects are specific for SAHA and hence the inhibition of xCT expression with reduced glutamate secretion was further examined in another experiment utilizing the HDAC inhibitor MS-275, which does not induce xCT suppression in malignant gliomas (Fig. 3A). Treatment of the tumor implanted brain slice cultures with 8 μM MS-275 did not lead to a significant reduction in peritumoral cell death. We found a cell death rate of 220% which was comparable to the cell death levels found in tumor implanted groups without treatment (Fig. 5C). These data demonstrate that SAHA can specifically reduce xCT-dependent cell death.

## Discussion

**Figure 6:**
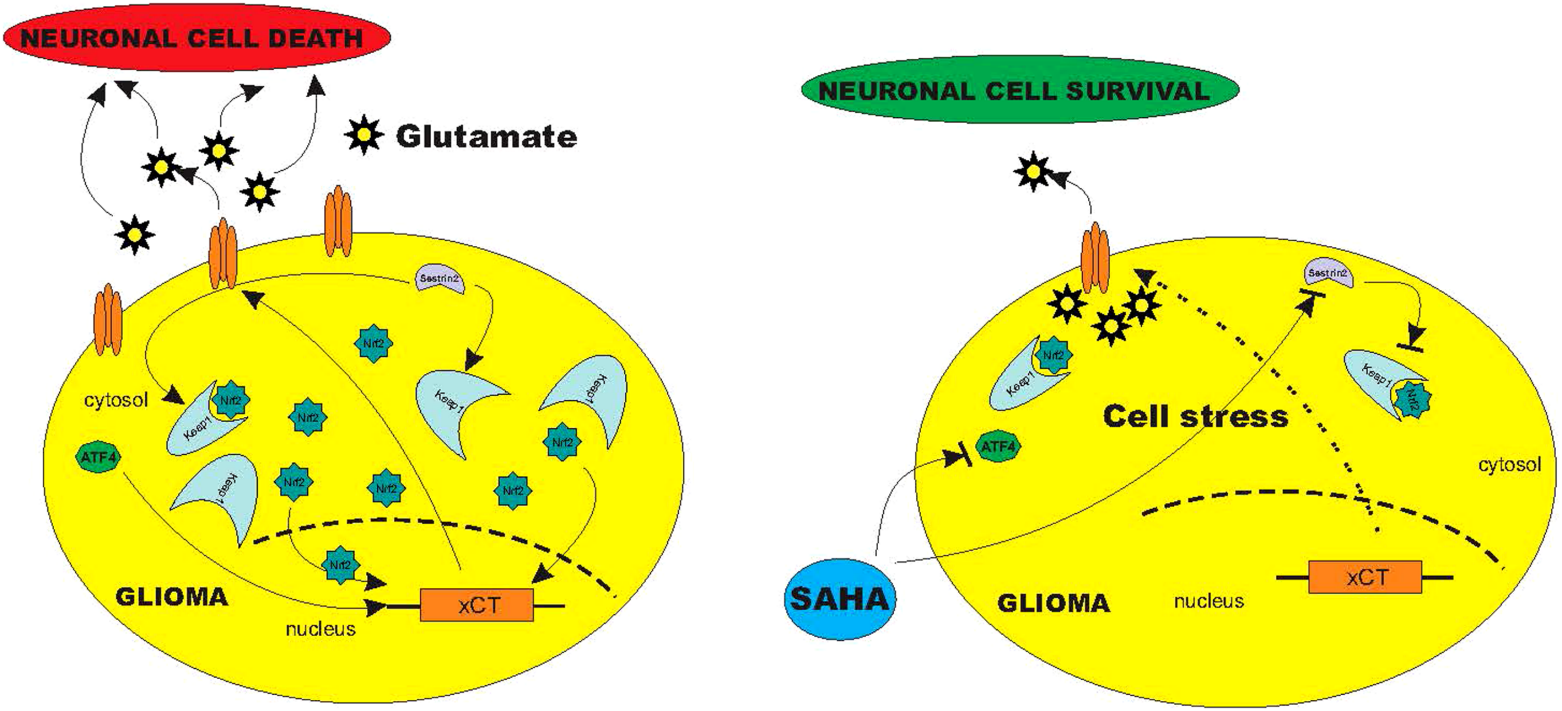
Schematic diagram of the SAHA induced blockage of glioma glutamate release. Summary of experimental data reveal that malignant brain tumors secrete neurotoxic concentrations of glutamate via xCT transporter, which is regulated by sestrin2 and ATF4. While sestrin2 controls Keap1 and Nrf2, ATF4 regulates xCT-expression directly on the promotor site. Treatment with SAHA affects ATF4 leading to a reduced xCT-expression with subsequent reduced glutamate release into the extracellular space. SAHA alleviates xCT-dependent cell death probably by this transcriptional pathway.

Although the identification of oncogenic target genes represents an important aspect in oncology, the results are often difficult to assess. Since these target genes are often ubiquitously expressed, the problem naturally lies in the ability to modulate them in a specific manner in cancer cells without disturbing generally the physiological homeostasis. We identified xCT as a target gene playing a decisive role in the progression of malignant gliomas^17^,^34^. Due to its ubiquitous expression, it falls under the restrictions attendant to target genes and would therefore have limited therapeutic scope. We have been able to show however that xCT can be selectively equilibrated through the HDAC-inhibitor SAHA as a glioma-specific target, so that xCT associated glioma-progression oriented changes are inhibited in the tumor microenvironment (Fig. 6). An important aspect here is the selectivity of HDAC-inhibitors for transformed cells, which can be traced back to the manipulation on the equilibrium between thioredoxin and ROS^9^. These properties render HDAC-inhibitors a matter of enormous interest in the treatment of malignant gliomas. They act selectively on transformed cells as opposed to conventional chemotherapeutic agents which act globally on the entire brain parenchyma^10^,^11^. The chance is therefore high to be able to target tumor cells and cancer stem cells hidden in apparently healthy brain parenchyma at a distance from the primary tumor bulk and are responsible for tumor recurrences too^18^. In addition our data show that SAHA as an HDAC inhibitor leads to increased ROS generation through the selective inhibition of the xCT transporter. This signifies that ROS activation through HDAC inhibitors occurs neither directly nor through xCT independent signaling pathways. This implies that the increase in ROS activity following SAHA treatment is a consequence of xCT induced cell stress. Despite the fact that selectivity for transformed cells cannot be dismissed, HDAC-inhibitors play further a role in non-oncogenic processes like in recovery of cognitive function in neurodegenerative disease or in aging processes^36^. This broader activity spectrum can be explained by the fact that the therapeutic dosage of SAHA for tumor cells lies significantly below that for non-transformed cells, so that the thioredoxin-protection of normal cells is only breached in higher concentrations. In our investigations, SAHA normalized the expression of xCT in all analyzed glioma cell lines and in primary human glioblastoma cultures as well, whereas the xCT-expression in healthy brain parenchyma was not affected. Correspondingly, xCT inhibition is sufficient for diminishing gliomas-derived glutamate release. The extent of glutamate alteration may not appear to be as dramatic as that of xCT expression since this change would reflect a more long-term effect of SAHA. Long term effects of SAHA are the induction of massive tumor cell death which affects many signalling cascades simultaneously. Thus, under such pleotropic conditions do secretome analyses not lead to pharmacologically specific results. In the long run, these effects nevertheless result in neuroprotection in the tumor microenvironment^17^,^37^. Decreased cysteine uptake can lead to reduced glioma cell proliferation. Here we demonstrated that treatment with SAHA over 4 days leads to inhibition of glioma cell proliferation, whereas for the biochemical assessment of the secretome we centered the analysis around a time window of 24 h^23^.

In this context, the adhesion molecule CD44v plays an important role due to stabling and regulating the xCT complex^38^. Noteworthy, selective deletion of a tumor-specific variant of the CD44 molecule from the complex, CD44v9, leads to loss of the xCT transporter from the surface of the tumor cells with consequent suppression of tumor growth. This additionally leads to increased expression of the cell-cycle inhibitor p21/WAF^38^ — a phenomenon similar to treatment with HDAC-inhibitors: the xCT expression is reduced and generation of p21/WAF increased^23^. In this manner, tumor cells are brought to cell-cycle arrest at G0-G1 and consecutively to apoptotic cell death^26^. We assume that the observed effect of SAHA-specific xCT-inhibition is not directly involved in the CD44v/xCT – p21/WAF axis. Firstly, although we achieved a reduction of alterations in the tumor microenvironment, i.e. neuronal cell death, inhibition of glioma proliferation could not be demonstrated. Furthermore, although in case of an MS-275 treatment — an HDAC-class 1 inhibitor — no additional inhibition of xCT-expression was observed, an increase in p21/WAF was seen. It can therefore be assumed that the xCT suppression observed under SAHA is not carried out through the p21/WAF-CD44v axis. This implies however, that an HDAC-selectivity for xCT clearly exists: SAHA inhibits the HDAC-classes I, IIb, IIc and IV, whereas MS-275 only inhibits class I^39^. These results imply that xCT-suppression under SAHA-treatment can be traced back at least to a specific HDAC class — the HDACs 1, 2, 3 and 8 (class I) can therefore be excluded as potential candidates with the HDACs 4, 5, 7, 9 (class IIa), 6, 10 (class IIb) and 11 (class IV) remaining open. A synergistic effect from several HDACs cannot be excluded either, although clarification of this aspect has little relevance for therapeutic application. This needs to be addressed solely in the event of future considerations for the development of even more selective HDAC-inhibitors for therapeutic application. The same can be said of xCT-controlled transcription factors, since xCT-expression is primarily regulated via Sestrin2 through the release of Nrf2 via the degradation of Keap1 activating the xCT-promotor^32^. Independent of this signaling pathway, xCT-expression is additionally regulated by the transcription factor ATF4^33^. The inhibition of ATF4 expression here results in a reduction of xCT expression^33^. We have investigated both signaling pathways following SAHA-treatment. Whereas the expressions of Sestrin2 and its subordinate players Nrf2 and Keap1 hardly underwent any alterations, SAHA-treatment led to pronounced inhibition of the transcription factor ATF4. We assume that the observed xCT-suppression is caused by reduced ATF4 levels. However, since the Sestrin2-signaling pathway is also affected by SAHA, a synergistic effect of SAHA on both signaling pathways cannot be excluded. The exact mechanism remains to be unraveled in future experiments (Fig. 6).

Another important question is whether specific HDAC-inhibitors impact on temozolomide non-responders. These patients account for approximately 65% of all glioblastoma cases^40^,^41^. Since the standard chemotherapeutic agent in the management of glioblastoma is temozolomide, a DNA-alkylating agent, the options for temozolomide non-responders are still poor^42^,^43^. An important candidate for this resistance is the DNA-repair enzyme O6-Methylguanin-DNA-Methyltransferase (MGMT). Patients with hypermethylation of the MGMT promotor profit the most from temozolomide-treatment^42^. Should MGMT be active, damage incurred to DNA by temozolomide is repaired, i.e. patients in which MGMT is in a non-methylated status profit little from treatment with DNA-alkylators^42^. In this context it has been shown that the adhesion-molecule CD44 is controlled via methylation processes and stabilizes and controls the xCT-complex^44^. In addition, it was been shown that the glutamate-receptor mGlu3 controls the expression of the MGMT-enzyme and can therefore impart resistance to chemotherapeutic agents^45^. Inhibition of mGlu3 receptors here leads to sensitization of tumor cells to temozolomide. Control over the MGMT-system through secreted glutamate via xCT therefore represents a realistic overlap of two systems, which prior knowledge indicated to be hitherto independent of each other. This implies that a change in the xCT-system could possibly lead to a change in the MGMT-status — a realistic scenario could therefore be that HDAC-inhibitors influence methylation processes as well, although to date it has been accepted that methylation modulates gene transcription independent of HDAC^46^.

### Notes Added in Proof

During the course of peer review and processing of our manuscript for publication, two papers have been published recently with impact to our findings. First, Deighton *et al*. reported that xCT gene regulation in mouse cortical neurons occurs activity-dependent in the absence of Nrf2^48^. This gene regulation is most likely due to ATF4 and AP1 driven xCT regulation.

Second, it has been recently shown that two small molecule inhibitors, erastin and sorafenib, can selectively inhibit the glutamate-cystine antiporter xCT (Dixon *et al*.)^49^. Whether these compounds also affect the transcriptional network for xCT will be an important subject in future investigations.

## Methods

### Chemicals

PI was obtained from Sigma-Aldrich Cell culture media and supplements were purchased from Biochrome. The xCT antibody was kindly provided by Dr. P. Kalivas (Medical University of South Carolina, SC, USA).

### Monolayer culture

Human (U87MG), rat (F98) and mouse glioma cell lines (GL261) were cultivated as described using Dulbecco's modified Eagle's medium (Invitrogen, Karlsruhe, Germany) supplemented with 1% penicillin/streptomycin (PenStrep, Invitrogen), 0.3% amphotericin B (Invitrogen) and fetal bovine serum as indicated (Biochrom, Berlin, Germany).

### RNA isolation and RT–PCR

RNA isolation was performed using the RNeasy Mini Kit (Qiagen, Hilden, Germany). RNA concentrations were determined using a NanoDrop ND-1000 spectrophotometer (Peqlab, Erlangen, Germany). For quantitative RT–PCR analyses, one step RT–PCR was performed using the QuantiTect SYBR Green RT–PCR Kit on a 7500 Real-Time PCR System (Applied Biosystems, Darmstadt, Germany). Each 20 ml RT–PCR reaction contained 10 ng total RNA. The comparative method of relative quantification was used to calculate the expression levels of each target gene relative to time- and solvent-matched controls. The RT–PCR specificity was verified by melting curve analysis. The following primers were used for quantification of xCT, Nrf2, Keap1, Sestrin2 and ATF4:

xCT forward primer: TGCTGGCTTTTGTTCGAGTCT; xCT reverse primer: GCAGTAGCTCCAGGGCGTA. Nrf2 forward primer: TCTGACTCCGGCATTTCACT; Nrf2 reverse primer: GGCACTGTCTAGCTCTTCCA. Keap1 forward primer: TTCGCCTACACGGCCTC; Keap 1 reverse primer: GAAGTTGGCGATGCCGATG. Sestrin2 forward primer: GGCACTTCCGCCACTCA; Sestrin2 reverse primer: TCAGGTCATGTAGCGGGTG. ATF4 forward primer: GGTTCTCCAGCGACAAGG; ATF4 reverse primer: TCTCCAACATCCAATCTGTCC. Beta-actin forward primer: GCTCCTCCTGAGCGCAAG; Beta-actin reverse primer: CATCTGCTGGAAGGTGGACA. Beta-actin was used as reference. Real-time RT-PCR was conducted with three technical repetitions for each gene and each RNA sample. Primer for xCT and CD98 were obtained from Qiagen: SLC7A11 (xCT): Hs_SLC7A11_1_SG; SLC7a11 (xCT): Rn_LOC310392_SG_1; Slc7a11 (xCT): Mm_Slc7a11_1_SG; SLC3A2 (CD98): Hs_SLC3A2_1_SG; SLC3a2 (CD98): Rn_Slc3a2_SG_1; Slc3a2 (CD98): Mm_Slc3a2_1_SG.

### Cell proliferation assay

Cell proliferation was measured using the 3-(4,5-dimethylthiazol-2-yl)-2,5-diphenyl-tetrazolium-bromide (MTT) assay. Briefly, glioma cells were seeded into 96-well plates (5000 cells per well; total volume 300 ml per well), treated with the test compound or solvent and analyzed after 96 h. Each experiment was repeated at least three times. Signals were quantified using a Tecan Safire2 microplate reader (Tecan, Zurich, Switzerland).

### Live image videomicroscopy and organotypic glioma invasion model (OGIM)

Brain slice cultures were prepared and maintained as described^47^. After decapitation, the brains of seven-day-old Wistar rats were rapidly removed and placed into ice-cold preparation medium. The brains were cut in 350 μm thick horizontal slices in preparation medium and cultured in humidified atmosphere at 35°C and 5% CO_2_ according to the interphase technique in culture medium. The medium was changed 1 day after preparation and every second day thereafter. Stably GFP-transfected F98 glioma cells [5000 cells] (p-EGFPN1 from BD Biosciences Clontech) were implanted within a total volume of 0.1 μl medium into the entorhinal cortex (layer II and III) one day after slice preparation. One day after implantation and every second day, glioma growth and invasion were evaluated using an inverse fluorescence microscope (BX51 microscope; Olympus). At day 5, all slice cultures were incubated with 1 μg/ml PI for 20 min followed by complete medium exchange in order to visualize irreversibly damaged cells and apoptosis.

### Glioma invasion, ROS imaging, and cell death analysis

Morphometrical analysis was performed using high power optical fields digitized with a CCD camera (Soft Imaging System) equipped to a BX51 microscope (Olympus) and imaging Software (analySIS, Soft Imaging System). Fluorescence-labeled cells as well as PI staining intensities were analyzed by an Olympus microscope (IX 70) equipped with a TRITC (excitation filter 520–550 nm, barrier filter 580 nm) and FITC (excitation filter 450–490 nm, band filter 520–550) narrow band filter, a CCD camera and image software. Cross talk and fluochrome filter leakage between channels was controlled and corrected using software (Lv3.1). For detection of intracellular peroxides, intracellular accumulation of reactive oxygen species (ROS) was visualized by 5-carboxy-2-dichlorodihydrofluorescein diacetate (DCFDA; Molecular Probes, Invitrogen) for 30 min at 37°C. Fluorescence measurements were performed on a fluorescence plate-reader at Ex λ 488 nm and Em λ 530 nm. Cell cycle analysis was performed on a FACS Calibur (BD Biosciences) equipped with an argon laser line and a red laser diode.

### Amino acid profiling of glioma conditioned medium

Cells were seeded in 6-well plates at a density of 75.000 cells per well in full DMEM medium. For the experiments at least three independent wells per cell line were used. After incubation overnight, the medium was changed to only DMEM without any supplements. After incubating for another 24 hours, medium was collected and measurement was performed by using high performance liquid chromatography (HPLC). Amino acids were analyzed by ion-exchange chromatography and post-column ninhydrin derivatization technique using a fully automated amino acids analyzer (Biochrom 30+, Laborservice Onken, Gründau, Germany). For the amino acid analysis, 100 μL of sample was deproteinised with 100 μL of 10% sulphosalicylic acids. 20 μL of this supernatant was then loaded by the autosampler into a cation-exchange resin-filled column.

### Ethics statement

Studies with human tissue were conducted in compliance with the Helsinki Declaration and approved by the Ethics Committee of the Friedrich-Alexander-University of Erlangen-Nuremberg. All patients gave written informed consent to participate in the study.

Animal killing was performed in accordance with the German Protection of Animals Act §4 paragraph one and three. The announcement of rat and mouse killing was approved by the designee for animal protection of the University of Erlangen-Nuremberg (TS-7/12).

### Statistical significance

Data from experiments were obtained from at least three independent experiments and n ≥ 3 if not otherwise stated. Statistical analysis was performed using MS Excel 2010 (Microsoft Corp., Washington, USA) if not otherwise described in the figure legends. The level of significance was set at P* < 0.05 according to the international conventions. Error bars represent ± S.D.

## Author Contributions

N.E.S. and I.Y.E. conceived and designed the experiments; I.M.L.W. performed the in vitro experiments and protein analyses; Z.F. and S.S. performed the mRNA analyses; amino acid profiling was performed by M.R.; N.H., M.B., N.E.S. and I.Y.E. analyzed the data and contributed to techniques and analysis systems; N.H., N.E.S. and I.Y.E. wrote and edited the manuscript.

## Supplementary Material

Supplementary Information Supplemental information
