# Supplementary material for: Histone deacetylases inhibition by SAHA/Vorinostat normalizes the glioma microenvironment via xCT equilibration
Source: Sci Rep. 2014 Sep 17;4:6226. doi: 10.1038/srep06226 (PMC4165982; doi:10.1038/srep06226)

**Supplementary Information**

**Title of manuscript:**

Histone deacetylases inhibition by SAHA/Vorinostat normalizes the glioma microenvironment via xCT equilibration

**Authors:**

Ines M.L. Wolf, Zheng Fan, Manfred Rauh, Sebastian Seufert, Nirjhar Hore, Michael Buchfelder, Nic E. Savaskan and Ilker Y. Eyüpoglu

**Supplementary information includes:**

Supplementary figures S1-S2

**Supplementary figure S1:** The full length blots in Figure 2.


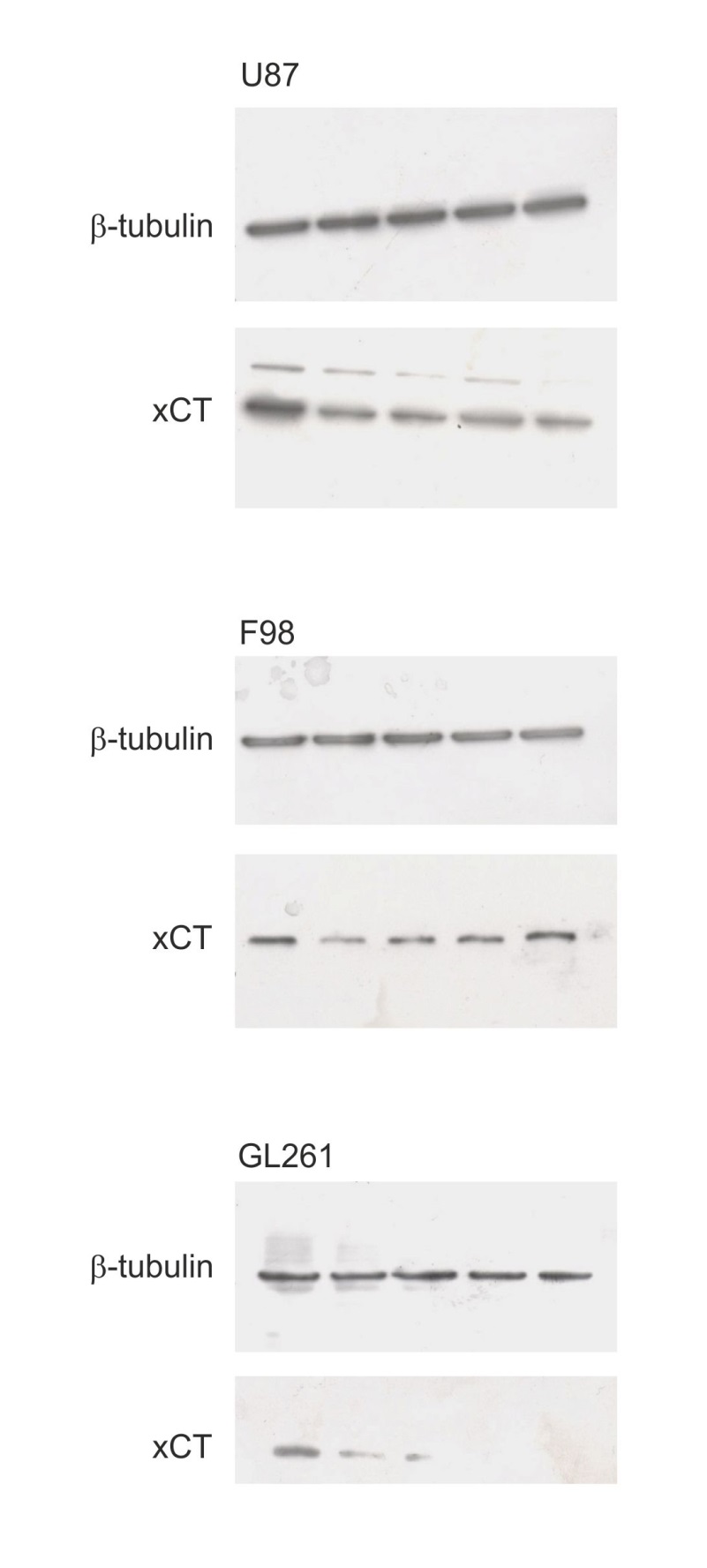


**Supplementary figure S2:** The full length blots in Figure 3.


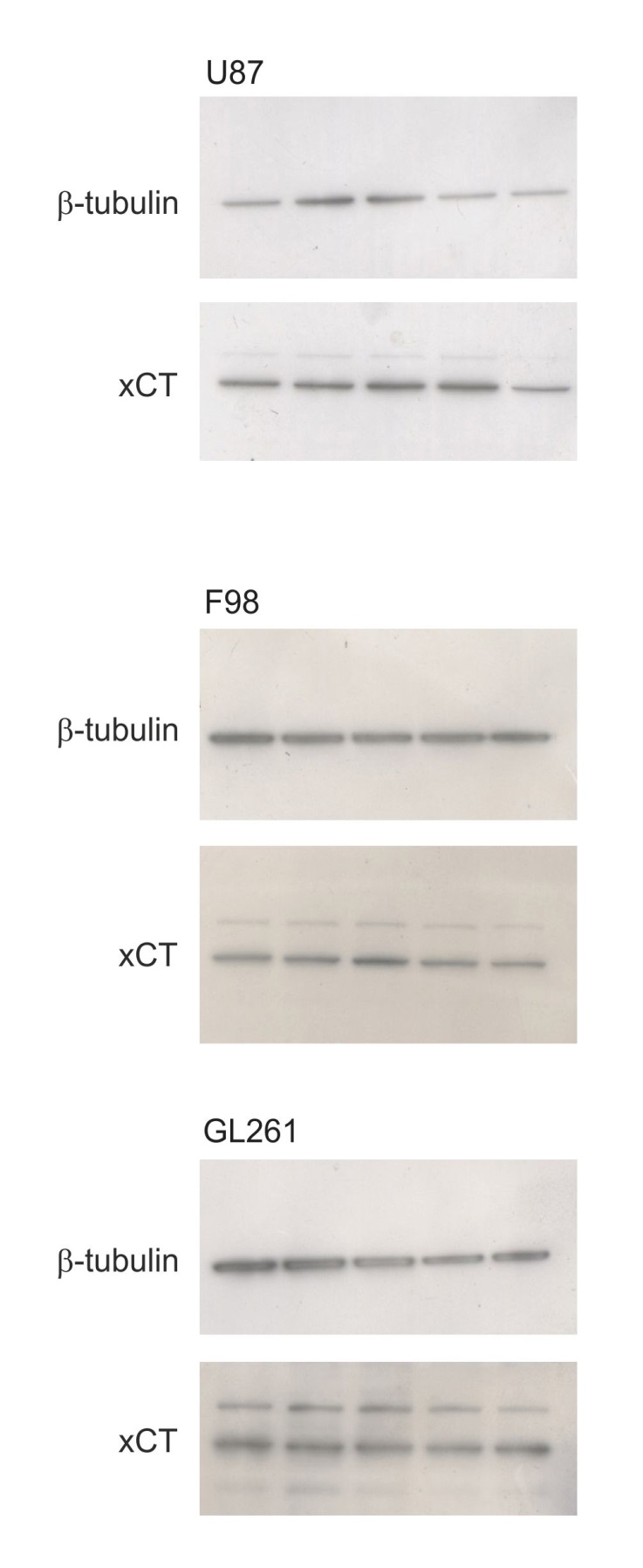

Supplement: Supplementary Information — Supplemental information [file srep06226-s1.doc]
